# Anatomical Location of the Common Iliac Veins at the Level of the Sacrum: Relationship between Perforation Risk and the Trajectory Angle of the Screw

**DOI:** 10.1155/2016/1457219

**Published:** 2016-12-18

**Authors:** Javid Akhgar, Hidetomi Terai, Mohammad Suhrab Rahmani, Koji Tamai, Akinobu Suzuki, Hiromitsu Toyoda, Masatoshi Hoshino, Sayed Abdullah Ahmadi, Kazunori Hayashi, Hiroaki Nakamura

**Affiliations:** Department of Orthopedic Surgery, Osaka City University Graduate School of Medicine, Osaka, Japan

## Abstract

*Purpose.* To determine the safety of transarticular surface screw (TASS) insertion and the anatomical location of the common iliac veins (CIVs) at the level of the promontorium.* Materials and Methods.* The locations of the CIVs on 1 mm computed tomography-myelography slices of 50 patients at the level of the promontorium and 20 human cadavers were investigated.* Results.* Among the patients, the left CIV was closer to the S1 anterior wall than the right CIV (mean distance: 5.0 ± 3.0 and 7.0 ± 4.2 mm, resp.). The level of the inferior vena cava (IVC) formation varied among the cadavers. The mean distance between the IVC formation and promontorium tip was 30.2 ± 12.8 mm. The height of the IVC formation and distance between the right and the left CIVs at the level of the promontorium were significantly correlated (*P* < 0.001).* Conclusion.* The TASS trajectory is safe as long as the screw does not penetrate the anterior cortex of S1. The level of the IVC formation can help to predict the distance between the right and the left CIVs at the level of the promontorium. The CIVs do not have a uniform anatomical location; therefore, preoperative computed tomography is necessary to confirm their location.

## 1. Introduction

**Figure 1:**
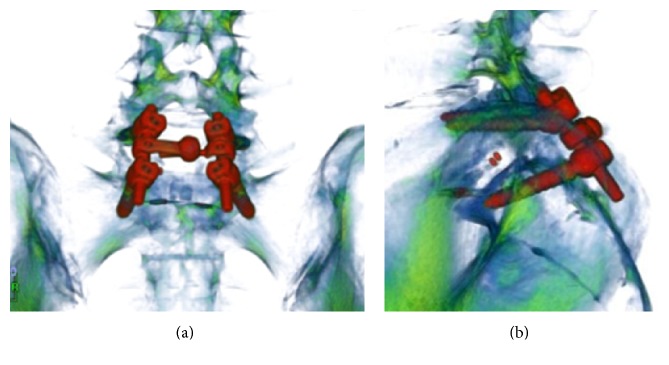
(a) Reconstructed coronal computed tomography image shows a combination of L5 cortical bone trajectory and S1 transarticular surface screw. (b) Reconstructed sagittal computed tomography image in the same patient.

Placement of screws in the sacrum is essential to ensure rigid fixation during instrumentation of the lumbosacral spine. Despite the availability of various screw implantation techniques for the sacrum, failure to achieve a safe, strong screw trajectory continues to be a significant clinical problem. Our center recently developed and implemented a novel sacral screw trajectory for S1 called transarticular surface screw (TASS) implantation. This technique can be combined with an L5 cortical bone trajectory screw and L5 pedicle screw for L5-S1 instrumentation and fusion (Figure 1).

A variety of techniques for sacral screw insertion to provide enhanced screw purchase have been described such as the cortical bone trajectory technique [1], the penetrating S1 endplate screw technique [2], bicortical and tricortical purchase [3], and the technique described by Luk et al. [4].

However, the safest S1 screw trajectory with which to obtain sacral fixation remains the main concern during screw implantation in the sacrum. Screw insertion toward the tip of the promontorium and S1 endplate to enhance screw strengthening is a cornerstone in many types of S1 screw implantation techniques [1, 3–8], including TASS implantation. However, one of the obstacles to safe screw insertion at this level is the close relationship of arteries, veins, and nerves with the anterior wall of the S1 vertebral body.

A thorough understanding of the anatomy of the structures at risk is a key to safe, successful implantation of screws in this region. Injury to the common iliac veins (CIVs) during S1 screw insertion is not a common complication, but it is serious and may be fatal [9, 10]. The close relationship of the CIVs with the anterior wall of the S1 vertebral body makes these veins susceptible to iatrogenic injury during S1 screw insertion [2, 9, 11]. The aim of the present study was to determine the anatomical location of the CIVs in front of the S1 vertebral body on reconstructed computed tomography-myelography (CTM) images and in human cadavers to determine the safest trajectory during TASS insertion.

## 2. Materials and Methods

After obtaining institutional ethics approval for this study, we investigated the anatomical location of the CIVs around the sacrum on 1-mm slices of reconstructed CTM images in 50 patients and 20 human formalin-embalmed cadavers to confirm the safety of TASS insertion (Table 1).

### 2.1. First Part of Study

Reconstructed CTM images of 50 patients (26 males and 24 females; mean age: 67.3 years; age range: 16–90 years) who underwent spine surgery in our institution were collected and analyzed by two investigators who used the same method and were blinded to each other's results.

**Figure 2:**
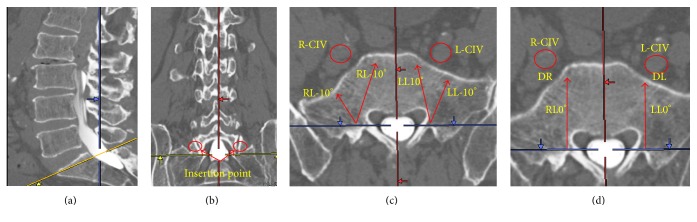
(a) Sagittal computed tomography image. The yellow line indicates the transarticular surface screw trajectory. (b) The red circle indicates the insertion point of the TASS in the coronal view. (c) R-CIV: right common iliac vein; L-CIV: left common iliac vein; RL10: trajectory 10° lateral to the insertion point; RL-10: trajectory 10° medial to the insertion point on the right side. LL10 and LL-10 are consistent with the same definitions on the left side. (d) RL0 trajectory straight and perpendicular to a horizontal line from the insertion point. DR and DL indicate the distance between the nearest wall of the CIV and the anterior wall of the S1 vertebra on the right and left side, respectively.

**Figure 3:**
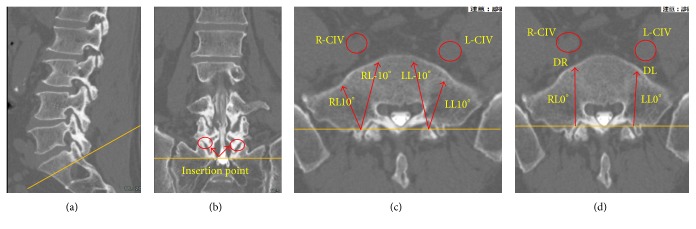
Sagittal coronal and axial computed tomography images from the trajectory parallel to S1 endplate. Abbreviations are as defined in Figure 2.

Two axial slices were meticulously prepared and printed by an expert spine surgeon; one slice was taken from the tip of the promontorium, and the other was taken 1 cm caudal to the tip of the promontorium, parallel to the S1 endplate (both slices included the TASS entry point). All measurements were performed on the above-mentioned printed axial slices (Figures 2 and 3).

### 2.2. Measurements

- The locations of the bilateral CIVs were determined in two planes. One plane included the TASS entry point (one-third the distance from the medial aspect of the superior articular process of S1 in axial and caudal level of pedicle, in sagittal location which is located within the articular surface and promontorium), and the other plane was parallel to the S1 endplate and included the TASS entry point (Figures 2 and 3).
- The angles of the CIVs from the insertion point of the TASS were measured.
- The shortest distance from the anterior wall of the S1 vertebra to the nearest wall of the CIV on both the right and the left sides was measured (Figures 2(c) and 2(d)).
- The distance from the insertion point of the TASS toward the promontorium and anterior wall of the S1 vertebra was measured in two planes and at three different angles (10°, 0°, and −10°).
- The height and body weight of each patient were measured to determine the presence of any correlation of the patient's height and weight with the distance of the right and left CIVs or any other parameters.

### 2.3. Second Part of Study

Twenty human embalmed cadavers (9 males and 11 females; mean age at time of death: 83.8 years; range: 63–103 years) that had been donated for medical education and research were used in the present study.

Each cadaver was placed in the supine position, and the abdomen was opened with a midline incision. All abdominal and pelvic organs were moved away.

**Figure 4:**
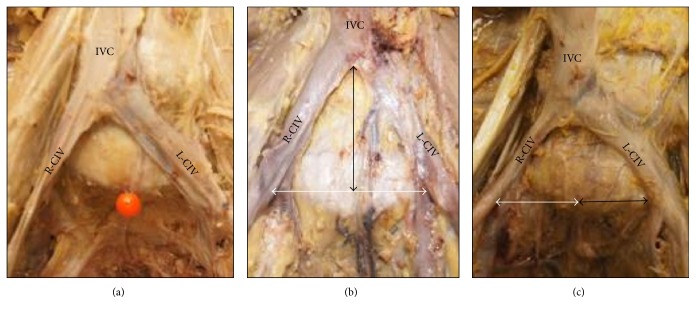
(a) Locations of the common iliac veins (CIVs) and inferior vena cava (IVC). The pin indicates the center of the promontorium. (b) The vertical arrow indicates the distance between the level of the IVC formation and tip of the promontorium. The horizontal arrow indicates the distance between the right and left CIVs. (c) The white arrow indicates the distance between the center of the promontorium and the medial wall of the right CIV (R-CIV), and the black arrow indicates the distance between the center of the promontorium and the medial wall of the left CIV (L-CIV).

To locate the center of the promontorium, we measured the width of the L5 vertebra with a digital caliper and divided it into two (Figure 4(a)). The center of the promontorium was then marked with a tiny needle.

### 2.4. Measurements

- The distance between the formation of the inferior vena cava (IVC) and the center of the promontorium was measured (Figure 4(b)).
- The distance between the center of the promontorium and the right and left CIVs was also measured (Figure 4(c)).

Upon completion of the measurements, the location of the IVC formation was marked and plain anteroposterior and lateral radiographs were taken of each cadaver to verify the location of the IVC radiographically.

The diameter of the median sacral vein was measured to identify cadavers with a dilated median sacral vein. The median sacral vein is an unpaired vein located at the medial aspect of the sacrum and accompanies the middle sacral artery to receive blood from the sacral venous plexus, emptying into the left common iliac vein. The median sacral vein was considered dilated when its diameter measured ≥2 mm [9].

### 2.5. Statistical Analyses

The mean and standard deviation were calculated for each measurement. Statistical analysis was performed using computer software (SPSS version 17; Chicago, IL, USA). A *P* value of <0.05 was considered statistically significant.

## 3. Results

### 3.1. CT Images

At the level of the promontorium, the left CIV was closer to the S1 anterior wall than was the right CIV (mean distance: 5.00 ± 3.00 versus 7.00 ± 4.28 mm, resp.; *P* = 0.016) (Table 2).

The average angle of the left and right CIVs ranged from 6° to 22° ± 7.0° and 2° to 20° ± 11.85°, respectively (Table 3).

The right CIV was located farther laterally from the center of the promontorium than was the left CIV (24.00 ± 4.65 versus 19.00 ± 6.44 mm, resp.; *P* = 0.001) (Table 4).

The mean distance between the right and left CIVs at the level of the promontorium was 43.00 ± 8.74 mm.

The average screw length in the TASS plane (trajectory toward the tip of the S1 cranial endplate) at three different angles (10°, 0°, and −10°) was equal on the right and left sides, and the overall screw length in the TASS plane at three different angles was longer than the trajectory parallel to the S1 endplate. However, the difference was not statistically significant (*P* > 0.05) (Table 5).

The mean height and weight of the patients were 159 cm and 60 kg, respectively. No correlation of the height and weight of the patients with the distance of the right and left CIVs at the level of the promontorium was found.

### 3.2. Cadavers

The CIVs lie within the connective tissues immediately in front of the S1 and L5 vertebral bodies.

The left CIV was located closer to the center of the promontorium than was the right CIV. The IVC formation was located cranial to the promontorium in all cadavers.

**Figure 5:**
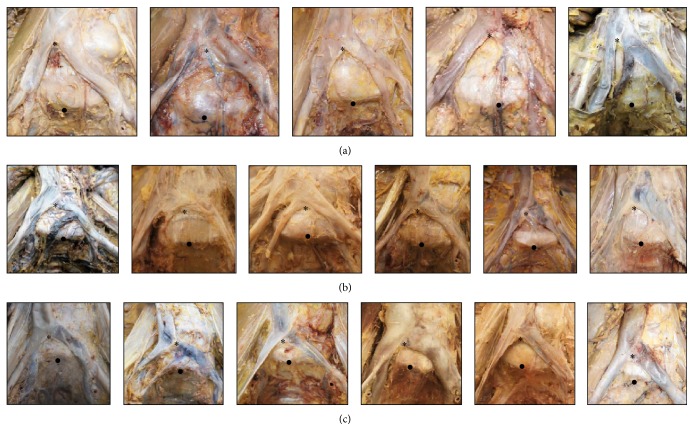
Three different levels of the inferior vena cava (IVC) formation ((a) high, (b) medium, and (c) low). ^*∗*^Level of IVC formation. ^●^Tip of promontorium.

**Figure 6:**
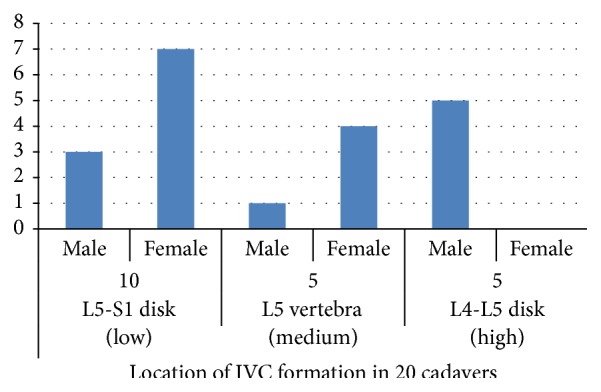
Distance between the level of inferior vena cava (IVC) formation and the promontorium in males and females.

Among the 20 cadavers, the IVC formation was located at the level of the L5-S1 disc space or L5 caudal endplate in 10 cadavers, at the level of the L5 vertebral body in 5 cadavers, and at the level of the L4-L5 intervertebral disc or L4 vertebral body in 5 cadavers (Figures 5 and 6).

**Figure 7:**
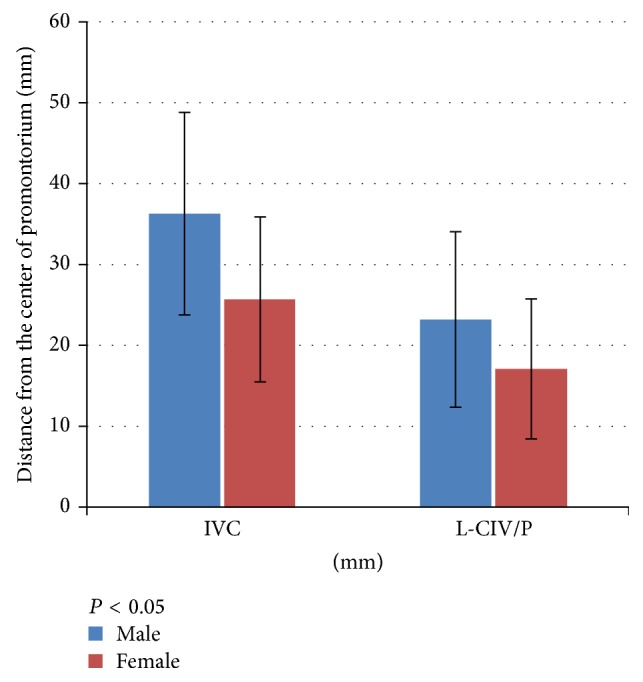
Location of the inferior vena cava (IVC) formation and the left common iliac vein (LCIV) from the center of the promontorium in males and females.

The level of the IVC formation varied among the different cadavers and by sex. A low location of the IVC formation was more common in female than in male cadavers (7 versus 3, resp.; *P* < 0.005) (Figure 6). The mean distance between the IVC formation and the center of the promontorium in male and female cadavers was 35.5 mm and 25.8 mm, respectively, and the mean distance from the center of the promontorium to the left CIV in male and female cadavers was 23.2 mm and 17.0 mm, respectively (*P* < 0.001) (Figure 7).

**Figure 8:**
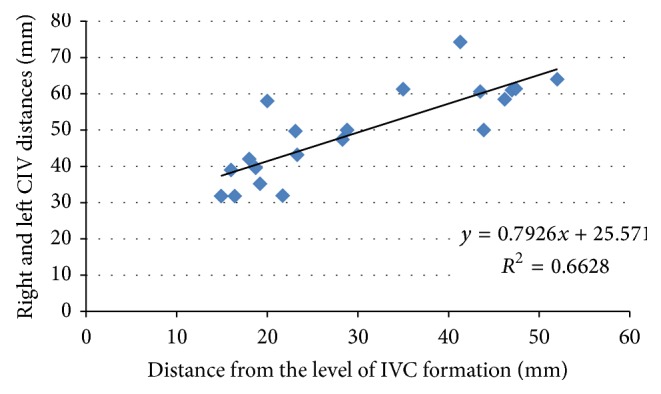
Correlation between level of inferior vena cava (IVC) formation and distance of right and left common iliac veins (CIVs) at the level of the promontorium.

There was a statistically significant correlation between the location of the IVC formation and the distance between the right and left CIVs at the level of the promontorium. In cadavers with a medium to high location of the IVC formation, the distance between the two CIVs at the level of the promontorium was wider than in cadavers with a low location of the IVC formation (*P* < 0.05) (Figure 8).

**Figure 9:**
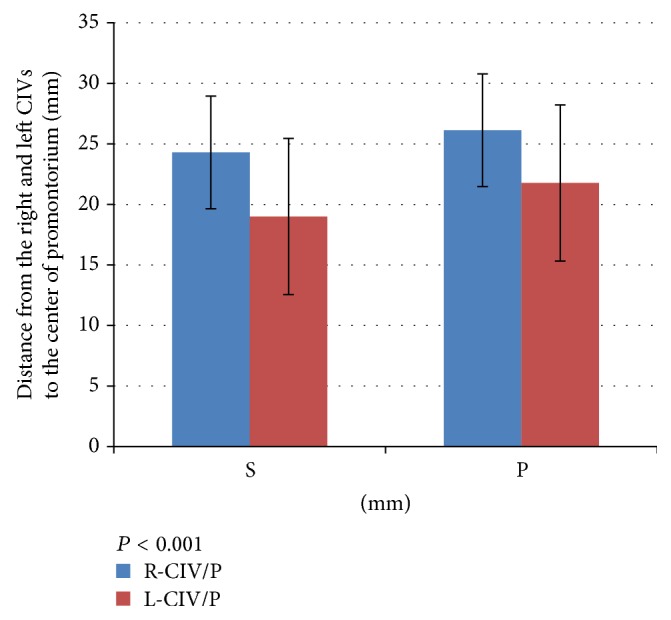
Distance from right and left common iliac veins (CIVs) to the center of the promontorium. S: level of the S1 cranial endplate; P: 1 cm caudal to the cranial S1 endplate.

The mean distance from the right and left CIVs to the center of the promontorium was 21.7 mm and 19.3 mm, respectively (*P* < 0.018) (Figure 9).

A dilated median sacral vein was observed in 4 of 20 cadavers (20%).

## 4. Discussion

The CIVs and internal iliac veins are located posterior and lateral to their corresponding arteries. The veins lie in the connective tissue immediately in front of the sacral ala, at the level of the first and second sacral vertebrae [12–14]. This is a typical anatomical location of these vessels (in front of the sacral bone). However, to describe the location of the CIVs and IVC formation based on their relationship with the center of the promontorium, a single acceptable location for the CIVs and IVC formation is needed, and such a location is difficult to define.

Iatrogenic injury to major vascular structures is a recognized complication of spine surgery [10, 15–18]. Such complications occur during posterior instrumentation of the spine in <1 of every 2000 operations [18, 19]. Injury to the aorta and iliac vessels can carry a mortality rate as high as 61% [19, 20].

In this study, the anatomical location of the CIVs, location of IVC formation, and distance between right and left CIVs varied in each individual (Table 6). Additionally, statistically significant differences in the correlation of the right and left CIVs with the center of the promontorium were observed between males and females. The existence of all of these differences is one of the main obstacles to designing a safe screw trajectory for S1.

The area between the right and left CIVs at the level of the promontorium seems to be safe if the tip of the screw penetrates the anterior wall of S1, but this distance is not uniform in all humans. We found a statistically significant correlation of the distance of the right and left CIVs at the level of promontorium with the location of the IVC formation. Therefore, we classified the IVC formation into three main groups: low formation (at the level of the L5-S1 disc space or L5 caudal endplate), medium formation (at the level of the L5 vertebral body), and high formation (at the level of the L4-L5 disc space and L4 vertebral body). In the low IVC formation group, the distance between the right and left CIVs at the level of the promontorium was significantly shorter than that in the high IVC formation group. This indicates that if the tip of the screw penetrates the anterior cortex of the sacrum during screw insertion, the chance of a CIV injury is higher in the presence of a low IVC formation than a high IVC formation.

In tricortical S1 pedicle screw purchase, Matsukawa et al. [3] recommend a screw orientation trajectory located as medially as possible in relation to the sacral midline. In this method, the anterior cortex of S1 is penetrated to achieve tricortical enhancement. We found that penetration of the S1 anterior cortex in each trajectory before evaluation of the location of the CIVs and IVC formation increases the chance of iatrogenic vessel injury because the location of these structures is not uniform in all humans.

Esses et al. and Ergur et al. strongly recommended avoiding anterior cortex penetration because of the risk of neurovascular injury [2, 9]. Our findings support those of other studies that recommended prevention of anterior cortex penetration during S1 screw insertion.

Mirkovic et al. [13] defined two safe zones for S1 screw placement: a medial safe zone and a lateral safe zone. The medial safe zone is bordered laterally by the sacroiliac joint, and its medial border is delineated by the lumbosacral trunk. The second zone lies between the sacral promontory medially and the internal iliac vein laterally. In the present study, the medial safe zone in female and low IVC formation was very narrow. Additionally, we found a dilated median sacral vein passing from the medial safe zone in 20% of cadavers, and this vein is highly susceptible to injury if the anterior cortex of S1 is penetrated by a screw.

Therefore, we strongly recommend avoiding penetration of the anterior wall of the S1 vertebra during S1 screw insertion in any trajectory, even in the center of the promontorium, because of the variable locations of the CIVs and dilated median sacral vein.

The risk of CIV injury is higher on the left than on the right side if the anterior cortex of S1 is accidently penetrated during the operation. Moreover, the risk of both right and left CIV injury during S1 screw implantation is higher in females than in males. The risk of CIV injury on either side is greater in patients with a low IVC formation than in those with high IVC formation.

Based on the findings of the present study, we present the following tips for safe TASS implantation:

- The tip of the screw should not penetrate the anterior cortex of the S1 vertebra.
- The length of the screw should range from 32 to 35 mm.
- A maximum screw length and low-risk trajectory can be achieved by insertion of screw at −10° toward the tip of the promontorium (directed 10° medially from the insertion point).
- CT evaluation before screw insertion is recommended to check the location of the CIVs, the level of the IVC formation, and selection of the appropriate screw length based on the axial and sagittal planes.

The information provided in this study will be useful for prevention of iatrogenic CIV injury during S1 screw implantation. Additionally, the mini-open anterior approach for L5-S1 fusion and instrumentation involves implantation of a cage between L5 and S1; therefore, a wide distance between the right and left CIVs is safer than a narrow distance. Spine surgeons can detect high-risk patients by checking the level of the IVC formation and distance of the right and left CIVs at the level of the promontorium based on the preoperative CT.

Some limitations of this study require acknowledgment. We used CTM images, which are less sensitive than CT angiography for detection of the CIVs. Additionally, embalmed specimens were used for this study, and we cannot exclude potential artifacts resulting from the embalming processes. The specimens were of relatively advanced age; thus, integrant spine scoliosis, anterior longitudinal ligament ossification, and other degenerative changes may have been present and might have changed some features of the CIV structures. Finally, we used Japanese cadavers, and our results might not be applicable to other races.

## 5. Conclusion

The TASS trajectory is safe as long as the anterior cortex of the S1 vertebra is not penetrated by the screw. The level of the IVC formation is a good indicator with which to predict the distance between the right and left CIVs at the level of the promontorium. The CIV does not have a uniform anatomical location; therefore, preoperative CT evaluation for confirmation of the CIV location is necessary.

## Figures and Tables

**Table 1:** Demographic characteristics of patients and cadavers.

Characteristics	CT images *n* = 50	Cadavers *n* = 20
*Age*		
Mean	67.3	83.8
SD	±13.7	±9.1
Minimum	16	68
Maximum	90	103
*Sex*		
Female	24 (48%)	11 (55%)
Male	26 (52%)	9 (45%)

**Table 2:** Distance between the anterior wall of S1 vertebral body and the nearest wall of common iliac veins on the right and left sides.

	Toward tip of S1 endplate (mm)		Parallel to S1 endplate (mm)	
	Mean	ICC (95% CL)	Mean	ICC (95% CL)
DR	8 ± 4.03	0.82 (0.71–0.89)	6 ± 3.71	0.81 (0.70–0.88)
DL	5 ± 3.01	0.84 (0.74–0.90)	5 ± 2.51	0.84 (0.74–0.90)
*P* value	0.016		0.0763	

DR: distance between the right CIV and anterior wall of S1 vertebral body on the right side; DL: distance between the left CIV and anterior wall of S1 vertebral body on the left side.

**Table 3:** Locations of the right and left common iliac veins based on vertical insertion screw trajectory.

	Trajectory toward tip of promontorium (°)		Trajectory parallel to S1 endplate (°)	
	Mean ± SD	ICC (95% CL)	Mean ± SD	ICC (95% CL)
A1(R)°	6.00 ± 6.58	0.74 (0.58–0.84)	9.00 ± 8.38	0.74 (0.58–0.84)
A2(R)°	22.00 ± 7.01	0.79 (0.66–0.88)	3.00 ± 8.51	0.79 (0.66–0.88)
A1(L)°	2.00 ± 9.73	0.78 (0.64–0.87)	3.00 ± 8.51	0.78 (0.64–0.87)
A2(L)°	20.00 ± 11.85	0.92 (0.87–0.95)	19.00 ± 9.28	0.92 (0.87–0.95)

A1R°: angle between vertical line (passing from insertion point toward anterior wall of S1) and medial border of the right common iliac vein; A2R°: angle between vertical line and lateral border of the right common iliac vein; A1L° and A2L°: same definitions but on the left side; SD: standard deviation.

**Table 4:** Distance between the right and left common iliac veins at the level of the promontorium.

Distance	Level of S1 cranial endplate (mm)	1 cm caudal to S1 cranial endplate (mm)	*P* value
R-CIV/L-CIV	43.00 ± 8.73	47.00 ± 15.90	0.015
R-CIV/P	24.00 ± 4.61	26.00 ± 8.12	1.091
L-CIV/P	19.00 ± 6.46	21.00 ± 7.14	0.018
*P* value (R-CIV/P versus L-CIV/P)	0.001	0.003	

Data are presented as mean ± standard deviation. R-CIV and L-CIV: distance between the right and left common iliac veins; R-CIV/P: distance between the right common iliac vein and the center of promontorium; L-CIV/P: distance between the left common iliac vein and the center of promontorium.

**Table 5:** Length of transarticular surface screw, toward promontorium and parallel to S1 endplate at three different angles.

	Toward tip of S1 endplate (mm)		Parallel to S1 endplate (mm)		
	Mean ± SD	ICC (95% CL)	Mean ± SD	ICC (95% CL)	*P* value
RL10°	31.00 ± 3.87	0.79–0.87	31.00 ± 3.56	0.79–0.87	>0.05
RL0°	36.00 ± 3.93	0.76–0.86	33.00 ± 4.15	0.76–0.86	>0.05
RL-10°	40.00 ± 4.45	0.71–0.83	35.00 ± 4.74	0.71–0.83	>0.05
LL10°	32.00 ± 3.88	0.88–0.93	31.00 ± 3.67	0.88–0.93	>0.05
LL0°	36.00 ± 4.23	0.72–0.83	34.00 ± 4.54	0.72–0.83	>0.05
LL-10°	41.00 ± 4.23	0.84–0.91	35.00 ± 4.54	0.84–0.91	>0.05

RL0: screw length from insertion point of transarticular surface screw toward anterior wall of S1 at an angle perpendicular to horizontal line passing through the facet joint on an axial computed tomography image; RL10: length of screw at 10° lateral trajectory toward anterior wall of S1 from insertion point on the right side; RL-10: length of screw at 10° medial trajectory toward anterior wall of S1 from insertion point on the right side; LL0, LL10, and LL-10 indicate the same information on the left side.

**Table 6:** Locations of common iliac veins and inferior vena cava formation in 20 cadavers.

Cadaver	R-CIV/P (mm)	L-CIV/P (mm)	R-CIV/L-CIV (mm)	IVC/P (mm)
1	37.1	27.3	64.2	52.3
2	33.3	28.4	61.3	47.4
3	19.2	20.1	39.4	16.3
4	40.2	18.3	58.2	20.6
5	19.1	23.2	42.3	18.3
6	29.3	2.8	31.8	14.9
7	32.8	14.6	47.4	28.3
8	30.6	30.8	61.4	47.4
9	36.1	24.7	61.3	35.2
10	28.9	16.3	43.2	23.3
11	26.3	23.7	50.3	43.9
12	24.2	36.4	60.6	43.5
13	26.1	5.8	31.9	21.7
14	32.5	7.1	39.6	18.7
15	27.2	31.5	58.5	46.2
16	42.3	32.3	74.3	41.3
17	24.3	6.6	31.8	16.4
18	21.8	13.3	35.2	19.2
19	38.3	11.4	49.7	23.1
20	27.1	23.5	50.2	28.8
Mean	29.7	19.8	49.5	30.2
Minimum	19.1	2.8	31.8	14.9
Maximum	42.3	36.4	74.3	52.3
SD	±6.66	±9.86	±12.46	±12.83

R-CIV/P: distance between the right common iliac vein and the center of promontorium; L-CIV/P: distance between the left common iliac vein and the center of promontorium; R-CIV/L-CIV: distance between the right common iliac vein and the left common iliac vein; IVC/P: distance between the level of inferior vena cava formation and the tip of promontorium; SD: standard deviation.
